# A case report of the rarest anal cancer: Basal cell carcinoma

**DOI:** 10.1016/j.amsu.2022.103291

**Published:** 2022-01-25

**Authors:** Jivatesh Tung, Bruce Lin, James Schlenker, Vlad V. Simianu

**Affiliations:** a Section of Colon and Rectal Surgery, Department of Surgery, Virginia Mason Medical Center, Seattle, WA, USA; b Section of Hematology-Oncology, Department of Medicine, Virginia Mason Medical Center, Seattle, WA, USA; c Section of Plastic Surgery, Department of Surgery, Virginia Mason Medical Center, Seattle, WA, USA

**Keywords:** Anal cancer, Basal cell carcinoma, Robotic surgery

## Abstract

A 69-year-old male truck driver with history of chronic anal fissures and facial basal cell carcinoma developed rectal bleeding and pain, and was diagnosed with a 5cm basal cell cancer of the anus with sphincter invasion. His workup entailed physical exam, CT and MRI which confirmed external and internal sphincter invasion without evidence of distant metastatic disease. After review of chemoradiation and surgical options, the patient elected to proceed with robotic-assisted abdominoperineal resection with end colostomy with complex local-tissue reconstruction. He is now two years out and disease free. While radiation and surgery have both been described in the literature as viable treatments, surgical resection may be the best option for patients with large lesions with sphincter invasion, who travel from afar and have occupational restrictions. This case highlights the importance of a multidisciplinary approach in assessing the patient with a rare disease process, presenting all viable options for treatment, and electing the optimal treatment through shared decision making.

## Highlights

• Basal (BCC) cell carcinoma makes up less than 1% of anorectal neoplasms.
• Radiation, cryotherapy, Moh’s surgery, local excision, and abdominoperineal resection can be used for anal BCC.
• Due to it’s rarity, we recommend a multidisciplinary approach for this pathology.
• Details of a robotic abdominoperineal resection with complex perineal closure performed are discussed.
• The highlight of this report lies in honoring patient's wishes and what treatment best fits their lifestyle.

## 1 Introduction

Basal cell carcinoma (BCC) of the anus is a rare lesion reported first by Bule and Brust in 1933 in a 53yo female who refused treatment [1]. There have been fewer than 200 cases in the literature since then. Though basal cell carcinoma can occur anywhere in the body and is classically described in the head and neck, it is estimated that <0.2% of BCC occurs in the perianal region [2]. Less than 1% of all anorectal neoplasms are basal cell [2]. It is important to distinguish this relatively benign carcinoma from other more aggressive anal carcinomas. Mohs’, wide local excision and radiation have all been described as treatments [[3], [4], [5]]. However, given the rarity of the disease, matching patient preferences with ideal treatment options is paramount. This work has been reported in line with the SCARE Criteria [6].

## 2 Patient information

A 69-year-old man presented with complaints of rectal bleeding and pain for two months. He had a history of chronic anal fissures and basal cell cancer of the face. His medical history was also remarkable for morbid obesity (body mass index of 33) and hypertension. He had a prior laparoscopic anti-reflux procedure, no family history of colorectal malignancy, and no history of tobacco use. His metabolic functional equivalents were greater than 4 [7]. He was a long-distance truck driver by trade.

## 3 Clinical findings, diagnostic assessment and timeline

**Fig. 1:**
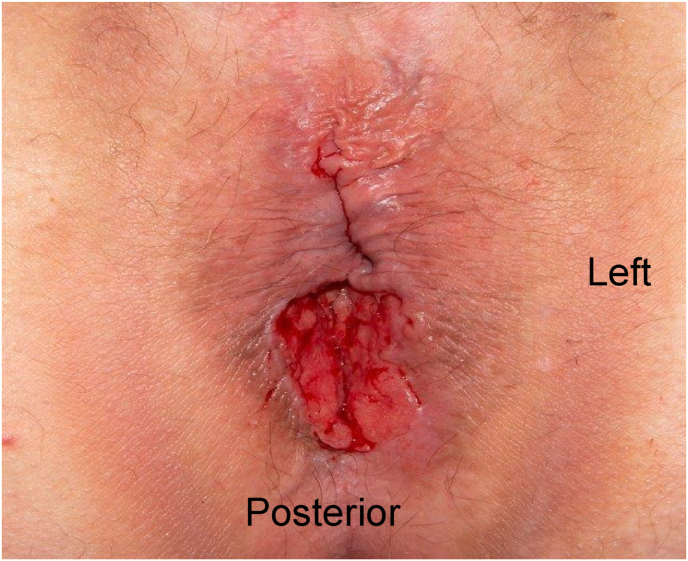
In lithotomy position, large posterior midline ulcerating anal mass.

Physical examination demonstrated a large, ulcerated fungating anal mass extending into the posterior midline of the anal canal (Fig. 1). Biopsy demonstrated an invasive basal cell carcinoma, nodular type, without lymphovascular or perineural invasion.

**Fig. 2:**
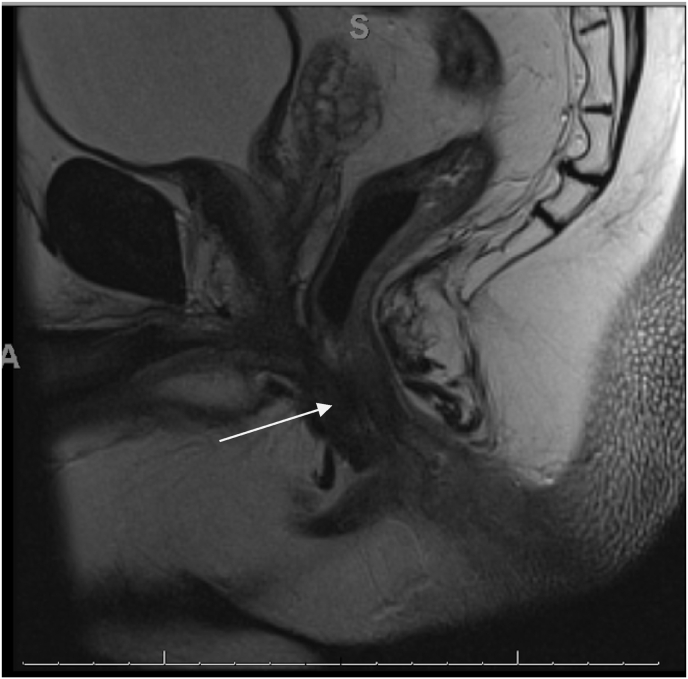
T2 MRI sagittal view of the mass shows invasion of the external sphincter complex.

Staging workup included CEA, colonoscopy, CT chest, abdomen and pelvis, and MRI Pelvis (rectal cancer protocol). He had a colonoscopy demonstrating 4 tubular adenomas in the colon and the posterior midline fungating mass extending into the anal canal, fixed to underlying muscle. MRI demonstrated invasion of internal and external anal sphincters with tumor dimensions of 4.8 × 3.1 × 1.3cm without adenopathy (Fig. 2). CT did not identify distant disease. CEA level was normal at 3.7 ng/ml. Findings and treatment options were reviewed at multidisciplinary colorectal cancer tumor board – including colorectal surgeon, radiation oncologist and medical oncologist. Due to involvement of sphincters, wide excision was not considered feasible. Through shared decision making, the patient elected to proceed with abdominoperineal resection. The patient's decision was influenced by his concern about the potential impact of bowel dysfunction after radiation therapy during his work as a long-distance truck driver.

Six weeks from his original presentation, he underwent abdominoperineal resection and perineal reconstruction.

## 4 Therapeutic intervention

He was managed on an enhanced recovery protocol receiving pre-operative mechanical and antibiotic bowel preparation, carbohydrate loading, immunonutritional supplementation and multi-modal analgesia using acetaminophen, gabapentin, and opioids. Beta-blocker and statin therapy was continued perioperatively. He had an optimal colostomy position marked by an enterostomal therapist prior to the operation. Preoperatively, he was given venous thromboembolism prophylaxis with 5000 units of subcutaneous heparin along with cefazolin and metronidazole for IV antibiotics. He was positioned in a modified lithotomy position with orogastric tube and foley catheter prior to incision. Case was performed by fellowship trained colorectal surgeon with 4 years of experience.

Pneumoperitoneum was established using veress needle technique and the abdomen explored laparoscopically without evidence of metastatic disease. Four robotic trocars (8mm x3, 12mm x1), as well as one 8mm AirSeal™ assist port were placed in horizontal orientation at the level of the umbilicus. A medial to lateral mobilization of the sigmoid colon was performed, and the superior hemorrhoidal vessels divided shortly after their take-off from the IMA. Lateral mobilization of the sigmoid colon was performed to allow adequate reach of the colostomy to the abdominal wall. A total mesorectal excision was performed, carried down to the levator muscles. The rectosigmoid junction divided with a robotic stapler, and a 19 mm French drain was secured to the rectal stump. Perfusion to the colostomy conduit was confirmed using ICG angiography which showed brisk blood flow in <60 seconds. The abdominal wall defect was made at the marked colostomy site and the sigmoid colon delivered through this area, and subsequently matured using 3-0 vicryl stitches along with 5 Brooking stitches.

The patient was then repositioned in the prone position, and an extra-levator perineal dissection was carried out circumferentially to include a wide margin on the posteriorly located BCC. The specimen was removed through the perineum and the previously secured drain left in the dependent pelvis. Closure of the perineal defect was performed by the plastic surgical team, using multiple layers of interrupted absorbable stitches to approximate the levator muscles, subcutaneous tissues, and skin.

**Fig. 3:**
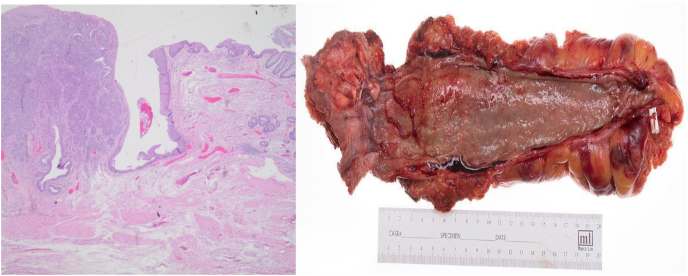
aTransition of normal, squamous epithelium to basal cell carcinoma. **3b.** Gross pathology specimen demonstrating the mass in the anal canal.

Post operatively, multimodal analgesia was employed with systematic diet advancement while minimizing upright sitting for the first 2 weeks after surgery. His postoperative course was complicated by ileus and he was discharged on postoperative day 11. The final pathology revealed a 6.3cm by 5.5cm by 0.5cm basal cell carcinoma with invasion into external and internal sphincter skeletal muscle with margins free of carcinoma and without perineural, angiolymphatic or lymph node invasion (Fig. 3A and B).

## 5 Follow-up and outcomes

Follow up was performed based on the NCCN guidelines for Anal Carcinoma with Digital rectal exam (DRE) and inguinal node palpation every 6 months. In addition, anoscopy and CT chest, abdomen and pelvis with contrast annually [8]. He was most recently seen two years post-operatively without any signs of BCC recurrence on exam or imaging.

## 6 Discussion

Differential for anal canal and margin tumors include squamous cell carcinoma, its precursor anal intraepithelial neoplasia (AIN) also known as Bowen's disease, adenocarcinoma, Paget's disease, melanoma and basaloid squamous cell carcinoma [9]. Rarest of all is basal cell carcinoma. While differentiating basal cell carcinoma from basaloid squamous cell carcinoma wouldn't have changed the treatment for our patient, they pose a significant diagnostic challenge to the surgical pathologist. Basaloid squamous cell carcinoma tends to be clinically more aggressive disease. Morphologic assessment along with immunohistochemical markers can reliably aid in distinguishing these lesions [2]. Staging recommendations for rare anal cancers follows recommendations for NCCN anal carcinoma with DRE, inguinal lymph node evaluation, anoscopy, CT chest, abdomen and pelvis and HIV testing [8].

Etiology of basal cell cancer in the anal region is unclear. Frequent association with trauma, prior radiation, and other skin lesions has been described in the literature [5,10], but was not the case for our patient. Interestingly, our patient had a history of basal cell carcinoma on his face. Basal cell cancer has been associated with mutations of P53 and inappropriate hedgehog signaling pathway activation in half of the cases [11,12]. Once present, the risk of distant spread from basal cell carcinoma is very low [9]. Most lesions have been reported to be less than 2 cm^2^ in overall area [9]. Our patient presented with a 4.8cm anal BCC, with staging imaging showing invasion of his sphincters. There is a dearth of literature describing radiation treatment in lesions this size however large lesions measuring 10 cms have been described to respond to radiotherapy [12]. Gibson et. Al review of 51 cases described treatment with radiotherapy, cryotherapy, immunotherapy, retinoid and chemotherapy, with radiotherapy being the most widely employed non-surgical option [9].

This patient was presented with both radiation and surgical options, and, in line with rare but reported literature, the tumor board recommendation leaned towards preferring radiation treatment in his case. Despite this, given concerns about post-radiation bowel dysfunction in up to 50% of the patients after pelvic radiotherapy [13] with his truck driving job, he elected for surgery. While the 5-year survival rate nears 100% in case reports, local recurrence is common and has been described up to 30% in the largest series [10]. Because recurrences may be treated with wide local excision or radiation, frequent surveillance examination is imperative. We have been following the patient every 6 months post-operatively.

This case highlights the importance of multidisciplinary approach in assessing the patient with a rare disease process and presenting all viable options for treatment and electing the optimal treatment through shared decision making. This patient has done well for the past two years with the elected approach without recurrence.

## Ethical approval

Not a study. Patient consent was obtained.

## Sources of funding

None.

## Author contributions

Dr. Tung- Data aquisition, manuscript writing, critical review, final approval.

Dr. Simianu- Data aquisition, manuscript writing, critical review, final approval.

Dr. Schlenker- manuscript writing, critical review, final approval.

Dr. Lin- manuscript writing, critical review, final approval.

## Registration of research studies

1. Name of the registry: N/A.

2. Unique Identifying number or registration ID: N/A.

3. Hyperlink to your specific registration (must be publicly accessible and will be checked): N/A.

## Guarantor

DR. Vlad SIMIANU, MD.

## Consent

Written informed consent was obtained from the patient for publication of this case report and accompanying images. A copy of the written consent is available for review by the Editor-in-Chief of this journal on request.

## Provenance and peer review

Not commissioned, externally peer-reviewed.

## Declaration of competing interest

Dr. Simianu has received travel, lodging, and educational support from Intuitive Surgical, Inc.
